# Supervised resistance exercise for women with ovarian cancer who have completed first-line treatment: a pragmatic study

**DOI:** 10.1007/s00520-023-07754-y

**Published:** 2023-04-26

**Authors:** Christelle Schofield, Robert U. Newton, Dennis R. Taaffe, Daniel A. Galvão, Paul A. Cohen, Tarek M. Meniawy, Carolyn J. Peddle-McIntyre

**Affiliations:** 1 Exercise Medicine Research Institute, Edith Cowan University, Joondalup, WA Australia; 2 School of Medicine, University of Western Australia, Crawley, WA Australia; 3 St John of God Hospital, Subiaco, WA Australia; 4 Sir Charles Gairdner Hospital, Nedlands, WA Australia; 5 School of Medical and Health Sciences, Edith Cowan University, Joondalup, WA Australia

**Keywords:** Ovarian cancer, Resistance exercise, Muscle mass, Muscle strength, Physical function

## Abstract

**Objectives:**

In ovarian cancer (OC), suboptimal muscle morphology (i.e., low muscle mass and density) is associated with poor clinical outcomes, yet little is known about the effect of interventions aimed at improving these measures. We investigated the effect of resistance exercise after first-line treatment on muscle mass and density, muscle strength and physical function, health-related quality of life (QoL), and pelvic-floor function in advanced-stage OC survivors.

**Methods:**

Fifteen OC survivors participated in supervised resistance exercise twice weekly for 12 weeks (in-clinic or by telehealth). Assessments included muscle mass and density (dual-energy X-ray absorptiometry, peripheral quantitative computed tomography), muscle strength (1-repetition maximum [1RM] chest press, 5RM leg press, handgrip strength), physical function (400-m walk, timed up-and-go [TUG]), QoL (QLQ-C30 questionnaire), and self-reported pelvic floor function (Australian Pelvic Floor Questionnaire).

**Results:**

The median age was 64 (range 33–72) years, 10 women underwent neoadjuvant chemotherapy and five underwent adjuvant chemotherapy. All participants completed the intervention (median attendance = 92%; range 79–100%). Post-intervention improvements were observed for whole-body lean mass (1.0 ± 1.4 kg, *p* = 0.015), appendicular lean mass (0.6 ± 0.9 kg, *p* = 0.013), muscle density (*p* = 0.011), upper and lower body strength (*p* ≤ 0.001), 400-m walk (*p* = 0.001), TUG (*p* = 0.005), and social and cognitive QoL domains (*p* = 0.002 and 0.007), with no change to pelvic floor symptoms (*p* > 0.05).

**Conclusion:**

In this study, supervised resistance exercise effectively improved muscle mass and density, muscle strength, and physical functioning without deleterious effects on the pelvic floor. Considering the prognostic value of these outcomes, larger studies are needed to confirm the benefits of resistance exercise in OC supportive care.

**Supplementary Information:**

The online version contains supplementary material available at 10.1007/s00520-023-07754-y.

## Introduction

About 75% of ovarian cancer (OC) cases are diagnosed at an advanced stage (III or IV) [1]. Standard first-line treatment for advanced-stage OC involves primary cytoreductive surgery and adjuvant paclitaxel/platinum-based combination chemotherapy or neoadjuvant combination chemotherapy and interval cytoreductive surgery [2]. Women at high risk of disease progression often receive bevacizumab, an anti-angiogenic drug, in combination with chemotherapy [2], and those with pathogenic germline or somatic variants in BRCA1/2 are eligible for maintenance therapy with a poly (ADP-ribose) polymerase (PARP) inhibitor [3]. As a consequence of the high treatment burden, women with advanced-stage OC often experience a range of treatment side effects [4]. Fatigue, peripheral neuropathy, insomnia, pelvic floor symptoms, and mood disorders often persist well after treatment completion and are associated with decreased health-related quality of life (QoL) [4, 5]. Treatment and care are complicated by the fact that most women diagnosed with OC are older [4], and many are already malnourished and have suboptimal skeletal muscle morphology (i.e., low muscle mass and density) at diagnosis, irrespective of high overweight and obesity rates [6].

In recent years, research has highlighted the important role of skeletal muscle in cancer outcomes. Both suboptimal muscle morphology and muscle function (i.e., low muscle strength and physical function) are associated with poor treatment and survival outcomes [7–9]. In OC, suboptimal muscle morphology is highly prevalent and correlates with more post-operative complications and chemotherapy toxicity [10] and increased mortality [11]. In a cross-sectional study conducted by our group, we observed significantly lower physical function and upper body strength in post-treatment OC survivors compared with age-matched controls [12].

Exercise has a beneficial effect on cancer-related health outcomes such as anxiety, depressive symptoms, fatigue, physical function, and health-related QoL [13]. Resistance exercise specifically has been shown to increase muscle mass and muscle strength during and after cancer treatment [14, 15]. To date, exercise studies involving women with OC are limited and consist mostly of home-based interventions of predominantly aerobic exercise [16]. There is a paucity of information regarding the effect of resistance exercise targeting potential prognostic markers such as muscle morphology and function in this cancer group. Thus, there are currently no research-based cancer-specific recommendations for clinicians or women with OC on the role of resistance exercise in OC treatment and care. A better understanding of the effect of targeted resistance exercise, in a supervised setting, on skeletal muscle morphology and function is needed to inform future research and guide the design and application of tailored exercise programs in clinical settings. Therefore, the primary purpose of this study was to investigate the effect of a 12-week supervised resistance exercise intervention on muscle morphology and function in advanced-stage OC survivors who have completed first-line treatment. A secondary aim was to investigate potential changes in health-related QoL and self-reported pelvic floor function from pre- to post-intervention.

## Methodology

### Setting and participants

The research design was a prospective single-arm study. Women with histologically confirmed stage III or IV epithelial OC who completed first-line surgical and chemotherapy treatment between four and 12 weeks prior to participation were eligible. Participants were recruited from Sir Charles Gairdner Hospital and St John of God Hospital, Subiaco, in Perth, Western Australia. Exclusion criteria were (1) age younger than 18 years, (2) participation in a structured, progressive resistance exercise program two or more times/week in the previous 3 months, (3) inability to understand and speak English, (4) inability to obtain approval from the treating medical oncologist or general practitioner, (5) on 10 mg or more per day of prednisolone (or equivalent of other steroid medication within 4 weeks of starting the study), (6) on any experimental anti-cancer therapy during or within 8 weeks of starting the study, (7) for patients on olaparib—needing a blood transfusion within the first 8 weeks of starting olaparib, (8) existing or suspected bone metastases, and (9) any illness or disorder that could put participants at risk during exercise testing or exercise training, as determined by their specialist or general practitioner. Potentially eligible women were screened for eligibility by phone, and a recruitment pack was mailed to eligible participants.

### Exercise intervention

The 12-week exercise intervention was delivered in an exercise clinic or by telehealth (online via the Internet) based on participants’ preferences. Participants undertook two supervised exercise sessions (either in-clinic or by telehealth) and one unsupervised home-based exercise session/week on non-consecutive days. All supervised exercise sessions were administered by the lead investigator (CS), an accredited exercise physiologist. Initial exercise prescription was based on each participant’s baseline functional and strength test results and choice of exercise delivery and adapted for limiting treatment-related side effects, such as peripheral neuropathy, and pre-existing comorbid conditions. Supervised exercise sessions consisted of a 5-min warm-up, 40–50 min of resistance training, as dictated by operational constraints, and 5 min of stretching to conclude the session. All exercise sessions included at least one multi-joint lower body exercise (e.g., leg press, sit-to-stand, and wall squat) and two multi-joint upper body exercises: a pull movement (e.g., seated row, rowing with an elastic band) and a push movement (e.g., chest press, push-ups on knees) (Supplementary Table 1). Additional upper- and lower-body resistance exercises were included in all sessions based on each participant’s capability and the equipment available. For telehealth sessions, resistance exercises mimicked the movement patterns of in-clinic exercises, but participants utilized body weight and equipment available at their homes for resistance. Participants completed two to three sets of eight to 12 repetitions of each exercise at a resistance that was perceived as moderate to hard, based on individual participants’ feedback [17]. At the end of each supervised session, participants rated their perceived exertion according to the Borg Rating of Perceived Exertion (RPE) 6–20 scale. Unsupervised home-based exercise sessions consisted of resistance exercises with body weight and equipment available at participants’ homes, and flexibility exercises. Additional specific rehabilitation exercises were prescribed for participants with pre-existing musculoskeletal conditions. Exercise intensity was gradually increased based on participants’ ratings of perceived exertion and symptom level (verbal feedback from participants regarding fatigue, neuropathic, or musculoskeletal discomfort or pain).

### Outcome measures

Outcome measures were assessed at baseline (week 0) and post-intervention (weeks 13 or 14) at the Edith Cowan University Exercise Medicine Research Institute, Perth, Western Australia. Demographic and health history data including age, marital status, education level, employment status, comorbidities, and current medications, as well as cancer-specific information, were acquired by self-report and medical record review. Height (m) and weight (kg) were measured to calculate the body mass index (BMI; kg/m^2^).

#### Muscle morphology

Whole-body and regional bone-free lean mass and fat mass were assessed by dual-energy X-ray absorptiometry (DXA, QDR-1500; Hologic Discovery A, Waltham, MA, USA). Appendicular lean mass was derived from the sum of upper-limb and lower-limb bone-free lean mass [18]. Muscle area (mm^2^**)** and muscle density (mg/cm^3^) of the lower leg were determined by peripheral quantitative computed tomography (pQCT; XCT-3000; Stratec Medizintechnik, Pforzheim, Germany).

#### Muscle strength and physical function

Upper and lower body muscle strength was assessed using a one-repetition maximum (1RM) chest press and a five-repetition maximum (5RM) leg press [19]. Isometric grip strength was measured with a Jamar handgrip dynamometer (Lafayette Instrument Company, Inc., Lafayette, USA). Relative strength for all strength measures was calculated as absolute strength divided by body weight. Physical function was assessed by the 400-m walk test as an indicator of mobility and cardiorespiratory fitness [20] and the timed up-and-go (TUG) test as an indicator of mobility and dynamic balance [21].

#### Patient-reported outcomes

Disease-specific health-related QoL was assessed using the European Organization for Research and Treatment of Cancer Quality of Life Questionnaire Core 30 (EORTC QLQ-C30) version 3.0. The QLQ-C30 incorporates five functional scales (physical, role, cognitive, emotional, and social), nine symptom scales (fatigue, pain, nausea and vomiting, dyspnea, insomnia, appetite loss, constipation, diarrhea, and financial difficulties), and a global health and QoL scale [22]. Scores range from 0 to 100, with higher values indicating a higher level of the construct (QoL or symptoms) [22]. A 10-point change is considered clinically important [23].

Self-reported pelvic floor function was measured with the Australian Pelvic Floor Questionnaire [24]. The questionnaire has four subscales to assess bladder, bowel, pelvic organ prolapse (POP) symptoms, and sexual function. As most participants indicated sexual inactivity, we did not calculate sexual function scores. Separate scores out of 10 were calculated for bladder, bowel, and POP subscales and summed for a combined symptom score out of 30 to provide an overall pelvic floor score. Higher scores indicate more symptoms [24].

### Statistical analysis

For a single-arm pre- and post-intervention study with alpha at 0.05 and 80% power, 15 participants provide the ability to detect an effect size of 0.8. Data were analyzed with SPSS Version 28 statistical software (IBM Corp., NY). Variables were assessed for normality using the Shapiro-Wilk test. Results for frequency data are presented as number/percentage and mean/standard deviation for normally distributed data, or median/interquartile range for non-normally distributed data. To determine differences from baseline to post-intervention, we used the paired samples *t*-test or Wilcoxon’s signed rank test, as appropriate, for continuous variables, and McNemar’s test for categorical data. All tests were two-tailed, with significance set at an alpha level of 0.05.

## Results

### Participant characteristics

**Fig. 1:**
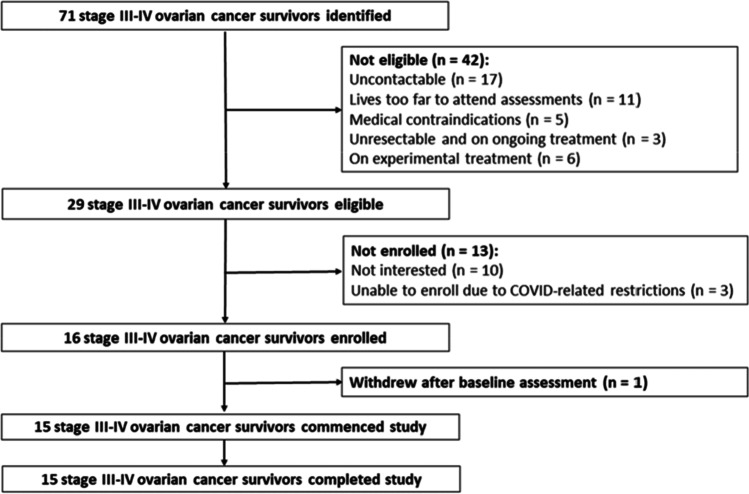
Participant flowchart

Between September 2019 and September 2021, 16 of 29 eligible women were recruited (Fig. 1). Fifteen participants started the intervention a week after their initial assessment. All fifteen participants completed the intervention. The median age was 64 (interquartile range, 46–68) years. Twelve participants (80%) had stage IIIC disease, and three participants (20%) had stage IVA disease. Ten participants (66.7%) received neoadjuvant chemotherapy and interval surgery, while five participants (33.3%) underwent primary surgery and received adjuvant chemotherapy. In addition to first-line surgery and chemotherapy, eight participants (53.3%) received further treatment. Most participants were partnered (73.3%), not currently working or retired (67.7%), non-smokers (60%), and had one or more comorbidities (67.7%) (Table 1). Overall, our sample is representative of the broader Australian OC population [25]. Participants were on average 60.7 days (range 28–91 days) post-primary chemotherapy treatment.

**Table 1:** Patient characteristics

	*n*	%
Age (years), median (IQR)	64 (46–68)	
BMI (kg/m^2^), mean ± SD	26.8 ± 6.3	
Disease stage		
III C	12	80.0
IVA	3	20.0
Treatment received		
Primary debulking surgery & adjuvant chemotherapy	5	33.3
Neoadjuvant chemotherapy & interval debulking surgery	10	66.7
Relationship status		
Partnered	11	73.3
Not partnered	4	26.7
Educational attainment		
Completed secondary school	8	53.3
Post-secondary certificate/diploma	2	13.3
University degree	5	33.3
Employment status		
Currently working part-time	5	33.3
Currently not working	3	20.0
Retired	7	46.7
Smoking status		
Non-smoker	9	60.0
Past smoker	6	40.0
Comorbidities		
0	5	33.3
1	5	33.3
≥ 2	5	33.3
Maintenance/additional treatment		
Bevacizumab	3	20.0
Olaparib	3	20.0
Bevacizumab and olaparib	1	6.7
Radiation	1	6.7

Abbreviations: *BMI*, body mass index; *IQR*, interquartile range; *SD*, standard deviation

### Exercise intervention

Twelve women (80%) chose to participate in person in the exercise clinic. The three women (20%) who opted for the online intervention indicated travel distance to the exercise clinics as the reason. The median attendance of the 24 scheduled supervised sessions was 92% (range 79–100%). Participants indicated reasons for missed sessions to be “not feeling well enough to exercise” (68% of missed sessions), non-medical appointments (16%), and family-related matters (16%). The mean rating of perceived exertion across all sessions was 13 ± 1 (i.e., somewhat hard). One participant was hospitalized for one night midway through the intervention with chest pain that was diagnosed as muscular pain, likely due to the intervention. No other adverse events were reported.

### Muscle morphology

**Fig. 2:**
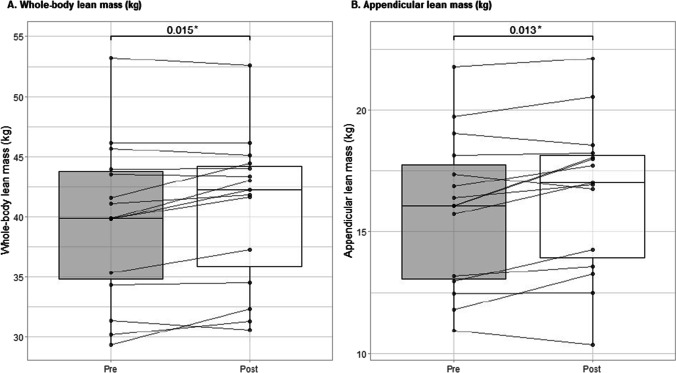
Individual changes from pre- to post-intervention—whole-body and appendicular lean mass. Lines and dots indicate individual data, with the box-and-whisker representation of the whole group.*p-value

From pre- to post-intervention, body weight increased by 1.4 ± 1.7 kg (*p* = 0.006) (Table 2). There was a significant increase in whole-body lean mass (1.0 ± 1.4 kg, *p* = 0.015) and appendicular lean mass (0.6 ± 0.9 kg, *p* = 0.013), with no significant change in fat mass or trunk fat mass. The muscle cross-sectional area of the lower leg did not significantly change (*p* = 0.176), although there was a modest increase in muscle density (*p* = 0.011). Individual changes in whole-body and appendicular lean mass from pre- to post-intervention are shown in Fig. 2.

**Table 2:** Pre- and post-intervention measures of body composition, muscle morphology, muscle strength, and physical function

	Pre-intervention	Post-intervention	*p*-value
Body weight	71.4 ± 17.5	72.8 ± 17.4	**0.006**
DXA			
Whole-body lean mass (kg)	39.7 ± 6.6	40.7 ± 6.2	**0.015**
Appendicular lean mass (kg)	15.9 ± 3.1	16.5 ± 3.2	**0.013**
Whole-body fat mass (kg)	30.1 ± 11.7	30.7 ± 12.0	0.139
Trunk fat mass (kg)	13.5 ± 6.9	13.9 ± 7.3	0.168
Whole-body fat percentage, %	40.1 ± 5.4	40.7 ± 5.6	0.713
pQCT			
Muscle area (mm^2^)	5444.3 ± 1069.6	5574.5 ± 1010.7	0.176
Muscle density (mg/cm^3^) (median and IQR)	73.1 (71.8–73.8)	73.5 (72.6–75.2)	**0.011**
Muscle strength Absolute strength			
1RM chest press (kg)	16.8 ± 6.2	22.7 ± 5.6	**< 0.001**
5RM leg press (kg)	56.6 ± 27.6	95.5 ± 39.5	**< 0.001**
Handgrip strength, R hand (kg)	24.4 ± 5.2	24.7 ± 6.0	0.667
Relative strength			
1RM chest press (kg)	0.2 ± 0.1	0.3 ± 0.1	**0.001**
5RM leg press (kg)	0.8 ± 0.4	1.3 ± 0.5	**< 0.001**
Handgrip strength, R hand (kg)	0.4 ± 0.9	0.4 ± 1.0	0.711
Physical function			
400-m walk (s)	310.0 ± 81.8	282.7 ± 60.6	**0.001**
Timed-up-and-go test (s)	7.8 ± 1.7	7.2 ± 1.5	**0.005**

Abbreviations: *DXA*, dual-energy X-ray absorptiometry; *pQCT*, peripheral quantitative computed tomography; *RM*, repetition maximum

Values are presented as mean (SD) unless otherwise specified

pQCT values, *n* = 14

Leg press *n* = 11, due to 4 participants being unable to complete the test at one or both time points

Chest press *n* = 12, due to 3 participants being unable to complete the test at one or both time points

Values in bold indicate statistically significant changes

### Muscle function

**Fig. 3:**
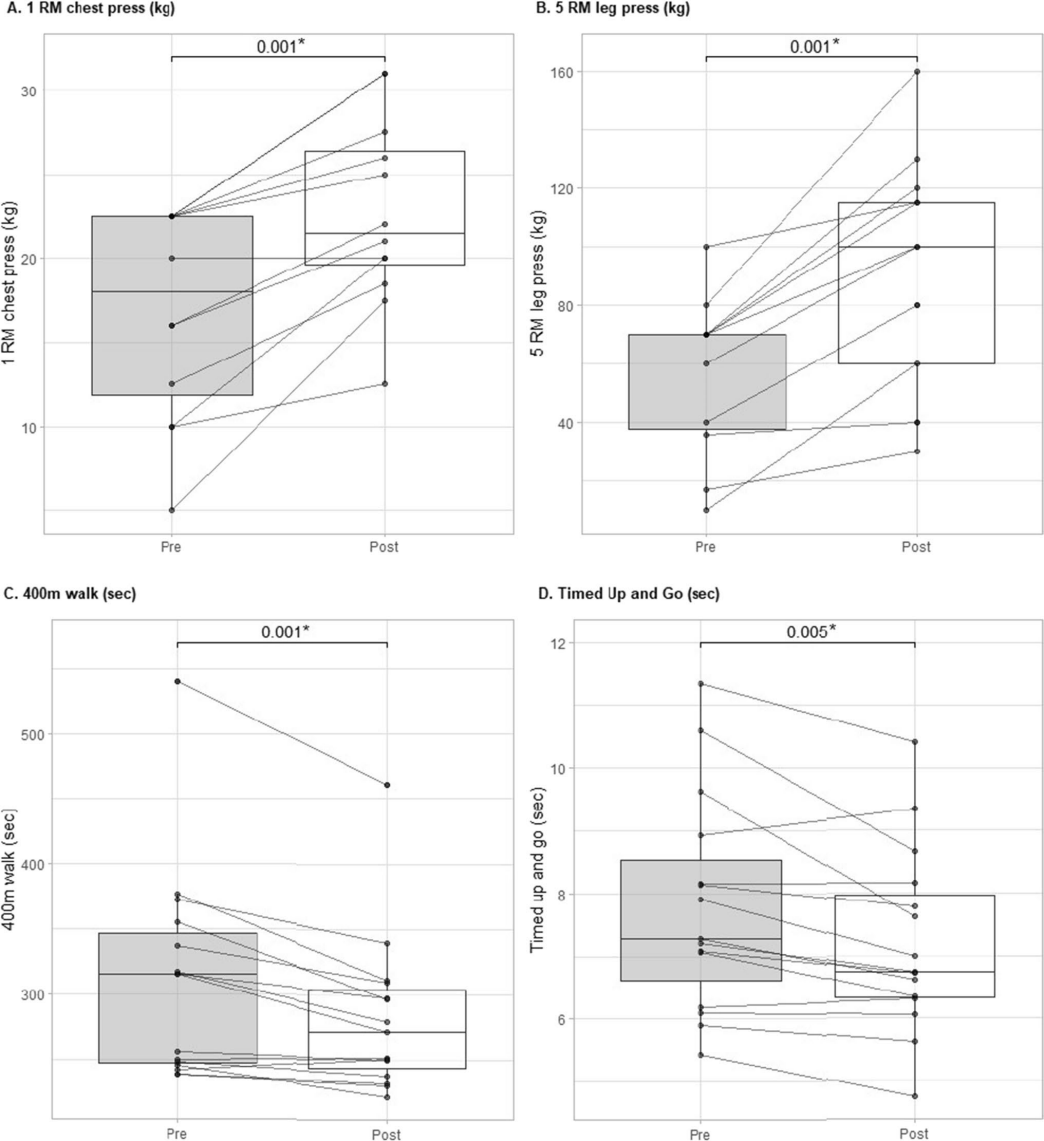
Individual changes from pre- to post-intervention—muscle strength and physical function. Lines and dots indicate individual data, with the box-and-whisker representation of the whole group. *p-value

Over the 12-week intervention period, there was a significant increase in upper body absolute strength (+ 5.8 kg, 95% CI: 3.6–8.1, *p* ≤ 0.001) and lower body absolute strength (+ 38.8 kg, 95% CI: 23.9–53.8, *p* ≤ 0.001) (Table 2). Upper and lower body relative strength also increased significantly (*p* ≤ 0.001), with no change in handgrip strength. The time to walk 400 m improved significantly (−27.4 s, 95% CI: 13.1–41.6, *p* = 0.001), as did the time to perform the TUG test (−0.6 s, 95% CI: 0.2–1.0, *p* = 0.005). Individual changes in muscle strength and physical function from pre- to post-intervention are shown in Fig. 3.

### Patient-reported outcomes

Of the five QLQ-C30 functional scales, social functioning and cognitive functioning improved significantly (*p* = 0.002 and 0.007, respectively) from pre- to post-intervention (Table 3). Of the nine symptom scales, only dyspnea displayed a statistically significant improvement from pre- to post-intervention (*p* = 0.034). Although changes in fatigue and global health and quality of life were not statistically significant, both scores changed by > 10 points, indicating clinically important improvements. In terms of self-reported pelvic floor function, mean bladder, bowel, and POP scores, as well as the pelvic floor score, were low pre- and post-assessment, with no statistically significant difference between scores at the two-time points (*p* > 0.05).

**Table 3:** Pre- and post-intervention patient-reported outcomes

	**Pre-intervention**	**Post-intervention**	***p*-value**
QLQ-C30 functional scales			
Physical functioning	93.3 (68.3–100.0)	90.0 (86.7–100.0)	0.198
Role functioning	100.0 (45.8–100.0)	100.0 (66.7–100.0)	0.075
Emotional functioning	79.2 (66.6–100.0)	75.0 (66.7–100.0)	0.765
Cognitive functioning	83.3 (66.7–83.3)	83.3 (83.3–100.0)	**0.007**
Social functioning, mean ± SD	58.3 ± 26.8	79.8 ± 14.9^#^	**0.002**
QLQ-C30 symptom scales			
Fatigue, mean ± SD	38.9 ± 27.5	27.8 ± 11.3^#^	0.110
Nausea and vomiting	0.0 (0.0–16.6)	0.0 (0.0–4.2)	0.180
Pain, mean ± SD	15.5 ± 16.6	21.4 ± 16.6	0.355
Dyspnea	16.7 (0.0–33.3)	0.0 (0.0–8.3)	**0.034**
Insomnia	33.3 (25.0–66.7)	33.3 (25.0–66.7)	1.00
Appetite loss	0.0 (0.0–33.3)	0.0 (0.0–33.3)	0.739
Constipation	0.0 (0.0–33.3)	0.0 (0.0–33.3)	0.705
Diarrhea	33.3 (0.0–33.3)	0.0 (0.0–33.3)	0.408
Financial difficulties	23.8 ± 33.2	14.3 ± 21.5	0.084
Global health & quality of life, mean ± SD	67.26 ± 18.03	77.4 ± 15.5^#^	0.051
Pelvic floor symptom scores			
Bladder score (/10)	0.9 (0.7–2.5)	1.0 (0.7–1.6)	0.153
Bowel score (/10), mean ± SD	2.0 ± 1.0	2.1 ± 1.0	0.775
POP score (/10), mean ± SD	0.0 ± 0.0	0.0 ± 0.0	1.000
Pelvic floor score (/30), mean ± SD	3.4 ± 1.8	3.1 ± 1.2	0.509
Sexually active, n (%)			
Yes	1 (7.1)	3 (21.4)	0.214
No	13 (92.9)	11 (78.6)	
Reasons for sexual inactivity:			
Not interested	7 (53.8)	7 (63.3)	
No partner	2 (15.4)	2 (18.2)	
Partner unable	1 (7.7)	0 (0)	
Vaginal dryness	1 (7.7)	0 (0)	
Other	2 (15.4)	1 (9.1)	
Not answered	0 (0)	1 (9.1)	
“How much do these sexual issues bother you?”			
Not applicable, I do not have problems	2 (15.4)	0 (0)	
Not at all	2 (15.4)	5 (45.5)	
Slightly	4 (30.8)	4 (36.4)	
Moderately	2 (15.4)	1 (9.1)	
Greatly	1 (7.7)	0 (0)	
Not answered	2 (15.4)	1 (9.1)	

Abbreviations: *POP*, pelvic organ prolapse, *QLQ-C30*, quality of life questionnaire core 30

Values are presented as median (interquartile range) unless otherwise specified

^#^Clinically important change (> 10 points)

Questionnaires completed by 14 participants

Values in bold indicate statistically significant changes

## Discussion

This study aimed to investigate the effect of supervised resistance exercise on muscle morphology and muscle function in advanced-stage OC survivors who had completed first-line treatment. There were two main findings. First, after 12 weeks of resistance exercise, there were significant increases in lean mass and muscle density, upper and lower body muscle strength, and physical function. Second, participants experienced clinically important improvements in global health and quality of life, fatigue, and social functioning, with no increase in pelvic floor symptoms.

We observed statistically significant increases in lean mass from pre- to post-intervention. Our finding is consistent with that of Lee et al. [26], who observed a significant increase in muscle mass measured by bioelectrical impedance after a 12-week resistance exercise intervention in a small sample of six OC survivors. In contrast, Cao et al. [27] found no significant difference in DXA-derived lean mass between OC survivors who participated in a 6-month aerobic exercise intervention and those who received usual care. The difference in findings is not surprising, considering that resistance exercise is superior to aerobic exercise in increasing skeletal muscle mass [28]. Evidence from a recently published meta-analysis confirms the efficacy of resistance exercise in increasing lean body mass in post-menopausal and elderly women [29]. In people with cancer, who often have low muscle mass already at diagnosis [7], resistance exercise has been proven effective to increase lean mass both during and after treatment [15]. Although our finding needs to be substantiated in a larger study, it underscores the importance of resistance exercise in recovering and increasing muscle mass after the completion of first-line OC treatment. Based on existing evidence [7], this may increase treatment tolerance for women requiring ongoing or future cancer treatment, with subsequent potential survival benefits.

In contrast to muscle mass, information on the effect of exercise on muscle density in people with cancer is limited, with some evidence that exercise during treatment preserves muscle density [30]. A recent meta-analysis demonstrates that exercise is effective in increasing muscle density in adults [31]. Resistance exercise specifically has been shown to increase muscle density in older adults without cancer [32] and in other clinical populations [33, 34]. Evidence suggests that low muscle density, but not low muscle mass, is associated with impaired physical function in older adults with cancer [35]. Furthermore, low muscle density appears to be a better predictor of mortality than low muscle mass in ovarian [10] and other cancers [36]. We observed a modest yet significant increase in muscle density after the resistance exercise intervention. Considering the prognostic importance of muscle density, the current finding, although warranting further investigation, adds important information about the potential therapeutic effects of exercise in cancer care.

Participants in our study experienced significant increases in physical function and upper and lower body muscle strength during the 12-week resistance exercise intervention. Only a few OC exercise studies have included measures of physical function or muscle strength [16]. Despite promising preliminary evidence for the beneficial effect of exercise on objective physical function and muscle strength after an OC diagnosis, more research is needed to inform the design and application of OC-specific exercise guidelines. To date, most exercise studies in OC have utilized only walking [16] or walking combined with a small resistance exercise component, which is often inadequately described [37]. Future research specifically investigating resistance exercise is crucial, especially considering evidence from breast cancer that exercise interventions that include resistance exercise as a single training modality, or in combination with aerobic exercise, are more beneficial for increasing muscle strength than aerobic exercise alone [38, 39].

We did not observe a change in handgrip strength after the 12-week resistance exercise intervention. While Moonsammy et al. also reported no improvement in handgrip strength after a 24-week multimodal exercise intervention [40], Lee et al. observed a significant increase in handgrip strength after 12 weeks of resistance training [26]. The different findings may be related to the timing of the different interventions and the detrimental impact of persistent peripheral neuropathy, a common side-effect of chemotherapy treatment [4], on potential improvement in handgrip strength. In contrast to our study and the study of Moonsammy et al., participants in the Lee et al. study had completed first-line treatment more than a year prior to participation and thus had more time to recover from chemotherapy treatment. Further, handgrip strength may not be a valid measure to evaluate changes in muscle strength following resistance-type exercise training [41]. Future studies involving women with OC during and/or after treatment should consider using measures other than handgrip strength to assess muscle strength.

Health-related QoL in OC survivors who had completed treatment is often negatively affected by persistent symptoms and side effects such as fatigue [4]. Further, decreased health-related QoL in this cancer group has been associated with lower physical activity levels [16]. We observed clinically important improvements for several health-related QoL components, specifically global health and QoL, social, cognitive, and role functioning, as well as for dyspnea and fatigue. Our findings are consistent with the results of a recent OC-specific systematic review indicating improved health-related QoL and fatigue after exercise training [16]. Although evidence indicates that exercise improves health-related QoL in this cancer group, further randomized controlled trials are needed to investigate the differential effect of different types of exercise interventions.

Importantly, patient-reported pelvic floor symptoms did not increase during the resistance training intervention. Although research investigating the effect of exercise on the pelvic floor is insufficient and often contradictory, current best evidence suggests that most physical activity does not harm the pelvic floor [42]. Our finding is consistent with evidence from a recent randomized controlled trial indicating no significant change in urinary incontinence in overweight, inactive women after a 12-week resistance training program [43]. Despite the high prevalence of pelvic floor dysfunction (PFD), especially urinary incontinence, in women with OC, rates of both pre- and post-treatment PFD do not differ significantly from those of women without cancer [5]. Most women in our sample experienced only a few mild pelvic floor symptoms (85% of women scored ≤ 5/30 for combined bladder, bowel, and POP symptoms). The variance in prevalence and severity of PFD indicates a need for screening at diagnosis and throughout the OC journey, with an appropriate referral for assessment and treatment when indicated. Our finding, albeit preliminary, suggests that OC survivors without severe PFD can participate in a supervised resistance exercise program without fear of harm to their pelvic floor.

This study investigated the effects of resistance exercise in advanced-stage OC survivors. We found that participation in a resistance exercise program shortly after the completion of first-line treatment can effectively improve muscle morphology and muscle function, both considered potentially prognostic for treatment and survival outcomes [7–11]. Despite homogeneity in terms of disease and treatment stage, our sample of women was diverse in terms of age, BMI, comorbidities, and physical functioning. Nonetheless, the intervention was well tolerated and well attended, with significant improvements in objective and patient-reported outcomes. Nevertheless, the study has important limitations that are worthy of consideration. The main limitation is the small sample size and the lack of a usual care control group. We cannot discount the fact that some degree of improvement in muscle morphology, muscle function, and health-related QoL may be due to natural recovery after first-line treatment completion. Because the intervention was delivered after the completion of first-line treatment, our findings may not be generalizable to women still undergoing cancer treatment. We acknowledge the possibility of recruitment bias, as women with an interest in, and previous history of exercise participation might have volunteered to participate. Also, women who were more physically deconditioned might have been more reluctant to volunteer due to a perceived inability to participate in resistance exercise. The pragmatic design of the study, which allowed women to choose between an in-clinic or online supervised intervention, may have affected the results. Women who participated in the online intervention had limited equipment available, which potentially limited muscle mass and muscle strength increases. Although we did not observe a significant difference in muscle morphology and function change between women who participated in the in-clinic versus online intervention (results not reported), we were likely underpowered to detect such differences. Future studies should investigate whether resistance exercise interventions delivered in-clinic versus online have differential effects on muscle morphology and function. Nonetheless, the study was specifically designed to allow for the online continuance of the intervention in the event of COVID-19-related restrictions and lockdowns. It is noteworthy that although women could choose between in-clinic or online participation, most women opted for the in-clinic option. This suggests a willingness to travel to appropriate exercise clinics to participate in supervised resistance exercise programs.

Although larger, controlled randomized trials are needed for confirmation, our findings suggest that supervised resistance exercise is beneficial for women who have completed first-line treatment for OC. Considering the growing interest in pre-habilitation for OC patients undergoing neoadjuvant chemotherapy, our findings also provide promise for pre-habilitation programs with a resistance training component.

## Conclusion

In this study, women with advanced-stage OC who recently completed first-line treatment experienced significant increases in lean body mass and muscle density, upper and lower body strength, and physical function after participating in resistance exercise. Clinically important improvements were also observed in fatigue, global health, and QoL, and social, cognitive, and role functioning. Importantly, participants did not experience an increase in pelvic floor symptoms. Although our findings need to be confirmed in larger trials, preliminary results show that targeted resistance exercise in a supervised setting is beneficial and should be recommended for women with advanced-stage OC.

## Supplementary information

ESM 1: Table S1 (DOCX 18 kb)
