# Supplementary material for: Supervised resistance exercise for women with ovarian cancer who have completed first-line treatment: a pragmatic study
Source: Support Care Cancer. 2023 Apr 26;31(5):304. doi: 10.1007/s00520-023-07754-y (PMC10132425; doi:10.1007/s00520-023-07754-y)
Supplement: Supplementary file 1 — Table S1 (DOCX 18 kb) [file 520_2023_7754_MOESM1_ESM.docx]

| **Supplementary table 1: Weekly resistance exercise programming** | | | | | |
| --- | --- | --- | --- | --- | --- |
|  | **Supervised Session 1** | | **Supervised Session 2** | | **Unsupervised session** |
|  | **In-clinic participants** | **Online participants** | **In-clinic participants** | **Online participants** | **Both groups** |
| Lower body exercises | Leg press^a, b^ | Sit-to-stand | Leg press^a, b^ | Sit-to-stand | One of:  - Sit-to-stand  - Wall squats  - Split squats  - Walking lunges |
|  | Supine glute bridge | Supine glute bridge | Supine glute bridge | Supine glute bridge | Supine glute bridge |
|  | Calf raises^c^ | Calf raises | Calf raises^c^ | Calf raises | Calf raises |
|  | One of:  - Wall squats  - Split squats  - Walking lunges  - Leg extension | One of:  - Wall squats  - Split squats  - Walking lunges | x | x | x |
| Upper body exercises | Seated chest press | Dumbbell press | Seated chest press | Dumbbell press | One of:  - Dumbbell press  - Push-ups |
|  | Seated row | One-arm bent-over row | Seated row | One-arm bent-over row | One of:  - One-arm bent-over row  - Standing rows with elastic  - Standing reverse flyes with elastic |
|  | x | x | One of:  Dumbbell press  Barbell press  Push-ups | Push-ups | x |
|  | x | x | One of:  One-arm bent-over row  Bent-over barbell row  Standing rows with cable/elastic | One of:  Standing rows with elastic  Standing reverse flyes with elastic | x |
|  | Dumbbell bicep curls | Dumbbell bicep curls | x | x | x |
|  | Tricep pushdown | Tricep kickbacks | x | x | x |
| Core exercise | x | x | Alternative arm-leg extension in four-point- kneeling /  Prone bridge | Alternative arm-leg extension in four-point- kneeling /  Prone bridge | Alternative arm-leg extension in four-point- kneeling /  Prone bridge |

Intensity of body weight exercises such as sit-to-stand, calf raises, push-ups, and prone and supine bridge was gradually increased according to each participant’s capability.

^a^ Leg press was initially replaced with supine single straight leg lifts for two participants with severe peripheral neuropathy.

^b^ All in-clinic participants did either the horizontal or angled leg press on different training days, depending on equipment availability.

^b^ Calf raises were initially replaced with seated ankle plantar and dorsiflexion for two participants with severe peripheral neuropathy.
